# Effects of a Specific Core Stability Program on the Sprint and Change-of-Direction Maneuverability Performance in Youth, Male Soccer Players

**DOI:** 10.3390/ijerph181910116

**Published:** 2021-09-26

**Authors:** Eric Brull-Muria, Jose Vicente Beltran-Garrido

**Affiliations:** 1 EUSES Escola Universitària de la Salut i l’Esport, Rovira i Virgili University, 43870 Tarragona, Spain; brullmuriae@gmail.com; 2 Department Education and Specific Didactics, Faculty of Humanities and Social Sciences, Universitat Jaume I, 12071 Castellón de la Plana, Spain

**Keywords:** core stability, youth, soccer

## Abstract

Although it is recommended to use sport-specific training programs to optimize performance, studies analyzing the effects of the core stability training with high levels of sport-specificity on athletic performance are insufficient and unclear. The objective of this study was to analyze the effects of the level of specificity of a CORE stability program on specific soccer actions. Fourteen youth players were randomly assigned to the specific core stability group (SCS; *n* = 7) or the general core stability group (GCS; *n* = 7). The eight-week intervention consisted of two weekly training sessions added to the usual soccer training. Both groups performed four CORE stability tasks. The SCS group followed the principle of sports specificity, while the GCS group performed CORE stability commons. Ten-meter linear sprinting (Sprint) and change-of-direction maneuverability (V-cut) were evaluated before and after the intervention programs. A statistically significant improvement was obtained in Sprint (*d* = 0.84 95% CI (0.22, 1.45), *p* = 0.008) and V-cut (*d* = 1.24 95% CI (0.52, 1.93), *p* < 0.001). At posttest, statistically nonsignificant differences were obtained between groups in Sprint (*d* = 1.03 95% CI (−0.25, 2.30), *p* = 0.082) and V-cut (*d* = −0.56 95% CI (−1.89, 0.78), *p* = 0.370). In conclusion, sprint and change-of-direction maneuverability were improved, but there was no superiority of any type of training.

## 1. Introduction

In recent decades, the concept of core stability (CS) has become fashionable, and central stability exercises are common elements of physical conditioning, training, and sports medicine programs [1,2,3,4,5,6]. However, the concept of CS is ambiguous in the scientific literature and the professional world [4,6,7,8,9], giving way to debates and confusion about training the stability, strength, power, and resistance of the core structures [2,10,11,12]. As suggested by Vera-García F. et al. [1], the definition of core stability is clearly linked to the context where it has been developed and used (i.e., biomechanical laboratories, rehabilitation clinics, and sports centers). This definition should start from the foundation developed in engineering and biomechanics, as well as the morphological and functional characteristics of the structures that form the CORE. In addition, it would be convenient to be able to apply this concept in different contexts such as sports training, fitness, and sports medicine, both in dynamic and static situations. Taking into account these considerations, the mentioned authors proposed the following definition [1]: the capacity of the osteoarticular and muscular structures, which is coordinated by the motor control system, to maintain or resume a position or trajectory of the trunk, when it is subjected to internal or external forces. If we apply the concept referred to training or sports medicine, the stability of the CORE can be understood as a physical quality, which is modifiable with training or rehabilitation, but it will always be context dependent.

In sports performance, conditioning programs of the central musculature are common since the core structures are responsible for stabilizing the trunk and transferring the forces of the central area of the body to the extremities in sports actions such as running, hitting, or throwing [2,5,13]. In addition, a rigid and stable core improves the mobility, speed, and distal limb performance of athletes [8,12,14,15]. Although these benefits are recognized in the literature, the results are unclear and present important limitations [1,3,6,7,9,10,11,14,16,17,18]. The suggested benefits of core training in soccer include reducing injury risk factors and improving performance in specific actions such as jumping [19], sprinting [20,21,22], and shooting [21] and in lower-limb asymmetries [23].

Where there seems to be a general consensus is to recommend CS exercises that follow the principle of sports specificity and that are functional, reproducing the usual movements and postures of athletes (i.e., standing Pallof press, chops, and lifts) [1,2,5,9,10,11,14,18,20,24,25,26]. Conventional stabilization exercises (i.e., bridges, bird dogs, and dead bugs) have not been shown to be effective in improving sports-specific performance [14,25].

Some of the recommended criteria to increase the sports specificity of the training load of the CS programs are based on the fact that the exercises are performed while standing up [1,7], involving three planes of motion [1,2,24,27,28]: reproducing specific movement patterns [1,2,28]; performing exercises at maximum movement speed [2,11,12,24] which is composed of an anti-rotational component [2,13,24] and which is predominantly unilateral [5,29,30]; and adding external resistance [2]. Although there are some studies that have been analyzed the effects of CS programs [31,32], the authors of the present study have not found any research that has analyzed the effects of a sports-specific CS program applying the cited recommended criteria, and it is reported as a limitation of some previous studies [14,24,28,33].

Power and speed have a key role in the most decisive situations of sport [34], which is why sprinting and change-of-direction actions are the most frequent in goal situations [34,35]. According to Barnes et al. [36], sprints in matches tend to be shorter and more explosive. In addition, 96% of the sprints of a soccer match are less than 30 m, and 49% are ≤10 m [35]. On the other hand, in a soccer match, there are between 35 and 38 changes in direction between 45 and 135° [37]. All this information reflects the importance of these specific actions on soccer performance and should be considered when assessing the effectiveness of training programs.

Due to the existing consensus in the literature when applying the principle of sports specificity, i.e., that the CS programs are generally cost-effective, time-efficient, and require cheap/minimal equipment to carry out so can be easily implemented at the community level, and the fact that no intervention study has designed a soccer-specific CS program, the objective of the study was to perform an exploratory analysis on the effects of the specificity of a *core stability program* on specific actions (sprint and maneuverability) in youth soccer players.

## 2. Materials and Methods

### 2.1. Experimental Approach to the Problem

To determine the effects of specific (SCS) and general (GCS) training on the ability to sprint and on the change-of-direction maneuverability, a randomized trial intervention of two groups was performed. The randomization of the study sample was carried out by a random number computer generator [38]. A stability training program of the CORE of 8 weeks (2 weekly sessions; at match day +2 days and match day −3 days) was added to the usual training of the soccer players during the competitive season. The sessions had 2 weekly sessions with a duration of 20 min each. Before and after the intervention period, linear 10-m sprint tests of 10 m (Sprint) and the change-of-direction V-cut test (V-cut) were performed [39,40].

### 2.2. Participants

A convenience sample of twenty-four youth, male soccer players from the same sub-elite team of the preferent youth league of Catalonia (Spain) voluntarily participated in the study. Potential participants were informed of the risks and benefits of the study before signing the informed consent form. The inclusion criteria of the study were as follows: (1) between 16 and 18 years old; (2) not performing regular CORE training; (3) not suffering from any injury that could impede the development of the intervention; (4) attending a minimum of 12 training sessions; and (5) attending the evaluation sessions.

The study was conducted according to the guidelines of the Declaration of Helsinki and approved by the Ethics Committee for Clinical Research of the Catalan Sports Council (protocol code 031/CEICGC/2021). Informed consent and assent were obtained from all subjects and their parents when the participants were minors.

### 2.3. Procedures

*Training intervention.* The training sessions were conducted in consensus with the rest of the technical staff of the team and following the recommendations of previous studies to facilitate the existence of a greater effect of the intervention on performance [14,20,41,42]. The intervention period lasted 8 weeks. The sessions lasted 20 min and lasted 2 weekly sessions. The interventions were performed as a warm-up prior to the field session [20,23,28,43]. The specific CS training was supervised by two instructors since the quality of the execution technique is a very important part of the intervention in multiplanar exercises [24]. The instructors were graduates in sports sciences and UEFA-licensed coaches with 5 years of experience in soccer training.

The training program was composed of 4 tasks: 2 tasks were performed in the transverse plane since core rotation is an important part of sports actions [13,24,44], 1 in the sagittal plane, and 1 in the frontal plane. In each session, 10 repetitions of a 10-s duration and 10 s of rest were performed [12,43,44].

Then, the following exercise was performed in vertical progression [45] and progressively until performing the 4. After the first 5 repetitions of the same exercise, one changed the sides and worked with the other hemisphere of the body. Before beginning each session, visual and verbal instructions were given to “activate the core so that no apparent movement was perceived in the central area of the body.”

During the course of a match, soccer players performed between 1000 and 1400 explosive actions of short duration, such as jumps, tackles, hits, turns, sprints, and changes in rhythm, with frequent changes every 4 to 6 s [35]. Other authors showed similar results, with 1290 action changes every 4.5 s [46] or 1200 activity changes every 3 to 5 s [47]. For this reason, it was proposed that every 5 s of each of the repetitions, the SCS group would produce an explosive action simulating the following specific movement patterns [35]: sprint, turn, acceleration, deceleration, change-of-direction, and jump. Depending on the type of task and progression, the same initial action was repeated (base exercises), the action was changed after the second 5 (progression 1), or dynamic stabilization was incorporated (progression 2).

*Training task of the SCS group.* For the creation of tasks in the SCS group, the 7 methodological criteria characteristic of the specificity of soccer in the performance stage were taken into account [1,2,5,7,10,11,12,13,18,24,26,27,28,30,33,44,48] and the reproduction of a specific sport action every 5 s [35]. The tasks, their progression toward multiplanar and dynamic stabilization actions, and the progression criteria can be observed in Table 1, Table 2, Table 3, Table 4 and Table 5. The participants progressed following an individualized approach (i.e., only when the participant showed proficiency in the task).

*Training task of the GCS group.* Based on the criteria for efficacy and safety in a systematic review by Vera-García et al. [25], bridges, bird dogs, and dead bugs are some of the most commonly used core stability exercises today. Among the bridges, the best known are the frontal bridge, the back bridge, and the lateral bridge. These exercises consist of maintaining the spine in a neutral position, that is, preserving the physiological curves when it is subjected to internal or external forces that test its stability, and they are mainly used in physical conditioning, physical education, and sports of initiation and recreation [25]. The bird dog and the lateral plank are two of the exercises called the Big 3 (curl-up, bird dog, and side bridge/lateral plank) for McGill [15]. The exercises proposed for the GCS group and the progression criteria can be observed in Table 6, Table 7, Table 8, Table 9 and Table 10.

*Performance test.* The selection of the tests took into account the methodology of structured training and the specific actions of soccer displacement that have a key role on performance [49,50], such as sprint actions and change-of-direction [34,35,36,37].

Before the tests, a standardized warm-up (10 min) was performed based on a study that investigated neuromuscular and athletic performance after training of the central musculature in young soccer players [21]. The first part common to all tests was performed, which consisted of a submaximal run (5 min). The second part was more specific (5 min) and involved submaximal exercises of the lower extremities. For the running tests, the specific part consisted of performing 10 body-weight squats, 3–5 jumps with countermovement, 2–3 short-distance submaximal linear sprints (10–15 m), and soccer-specific technical exercises (jumps, passes, running with the ball, and dribbling). For the change-of-direction test, the jumping actions were replaced by changes of direction, braking, and acceleration.

*Linear sprint measuring 10 m.* The sprint action was evaluated with the 10-m sprint test. The sprint time was recorded as fast as possible using photoelectric cells (Chronojump BoscoSystem, Barcelona, Spain) [51]. The participants started from a static position with one leg forward, according to preference, 1 m before the starting line [52]. The photocells were set up at a height of 0.64 m (approximately at knee height) [53], placed in a straight line, and the recording began when the first photocell was crossed. The time was measured with a precision of 0.01 s.

*Change-of-direction maneuverability.* The action of the change-of-direction maneuverability was evaluated with the 25-m test with 4 changes of direction of 45° every 5 m [39,40]. The time to sprint the 25 m with direction changes as fast as possible was recorded using photoelectric cells (Chronojump BoscoSystem, Barcelona, Spain) [51]. The participants started from a static position with one leg forward, according to preference, behind the starting line. Photocells were placed following the indications of Gonzalo-Skok et al. [40]. The recording began when the first photocell was crossed. For the attempt to be considered valid, the players had to cross the line, drawn on the ground, with one foot completely for each change-of-direction.

All tests were performed with the regulatory soccer boots of each participant. Three attempts were made for each test, leaving 5 min of rest between attempts. The best attempt was selected for subsequent statistical analysis. For familiarization with the tests, 2–3 previous attempts were made at a progressive intensity. Testing sessions were carried out with temperature and humidity conditions of 19 °C and 0%, respectively.

### 2.4. Statistical Analysis

The normality of the distribution of the data was checked using the Shapiro–Wilk test, and QQ plots were generated. The homoscedasticity was checked using Levene’s test. Data not following a normal distribution were log-transformed [54] before further analysis. Test reliability was examined by the coefficient of variation (CV) and a 2-way random intraclass correlation coefficient (ICC) with absolute agreement and 95% confidence intervals (CIs). Acceptable CV values were considered when CV ≤ 10% [55,56]. The ICCs were interpreted as follows: ICC <0.50 = poor, 0.5–0.74 = moderate, 0.75–0.9 = good, and >0.9 = excellent [57].

To assess between-group differences across the performance test scores, analysis of covariance (ANCOVA) using baseline values as a covariate was used. When a significant difference was found between the groups, Tukey’s post-hoc tests were used to determine the source. This approach is recommended when analyzing randomized trials with baseline and follow-up measurements, as it compensates for differences in baseline values [58]. To examine within-group changes from pretest to posttest, paired sample t-tests were employed. Effect sizes were calculated using Cohen’s *d* to further quantify between-group and within-group differences following the intervention. Effect sizes were interpreted as follows: <0.2 = trivial; 0.2–0.6 = small; 0.6–1.2 = moderate; 1.2–2.0 = large; and >2.0 = very large [59].

The level of significance was set at 0.05 for all tests. All statistical analyses were performed using Jamovi for Mac (version 1.8.4; JASP The Jamovi Project (2021), Sydney, Australia, retrieved from https://www.jamovi.org, accessed on 9 June 2021) and IBM SPSS Statistics for Mac (v.25, IBM, New York, NY, USA).

## 3. Results

**Figure 1:**
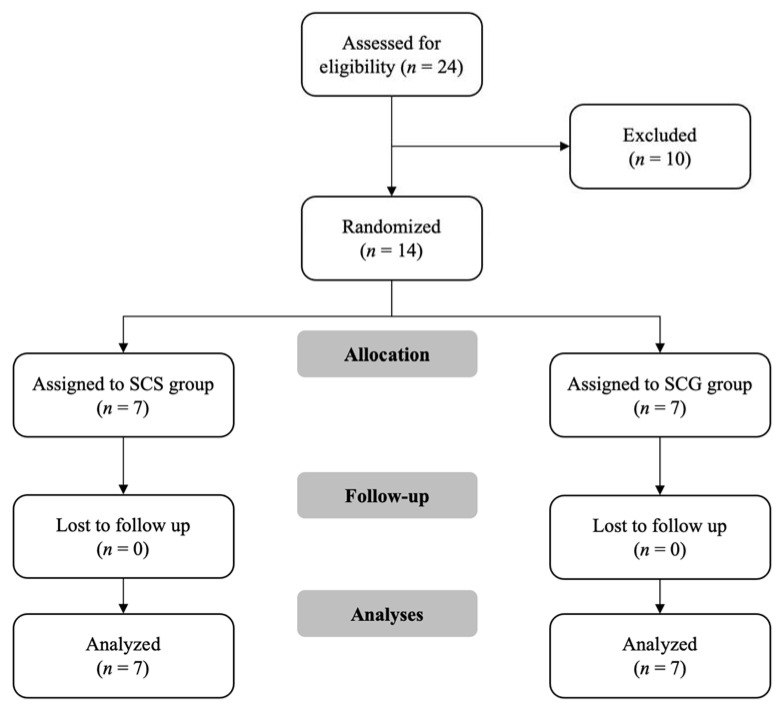
Consort diagram illustrating the flow of participants through the study. SCS: specific CORE stability intervention group; GCS: general CORE stability intervention group.

A consort diagram depicting the flow of participants throughout the study is presented in Figure 1. Initially, 24 male soccer players were screened, with 16 meeting all inclusion criteria. Three subjects were excluded for injury status; one subject was excluded because they were currently performing a CORE training program; four subjects were excluded because they were not assisted with the assessment sessions; and two participants decided to leave the soccer team. Finally, both training interventions were performed by 14 participants who attended 100% of the sessions (100% adherence). The baseline characteristics of the participants are presented in Table 11.

The reliability data of the test scores are presented in Table 12. Each test had acceptable between-trial consistency with all CV values <10% and moderate to good ICCs.

**Figure 2:**
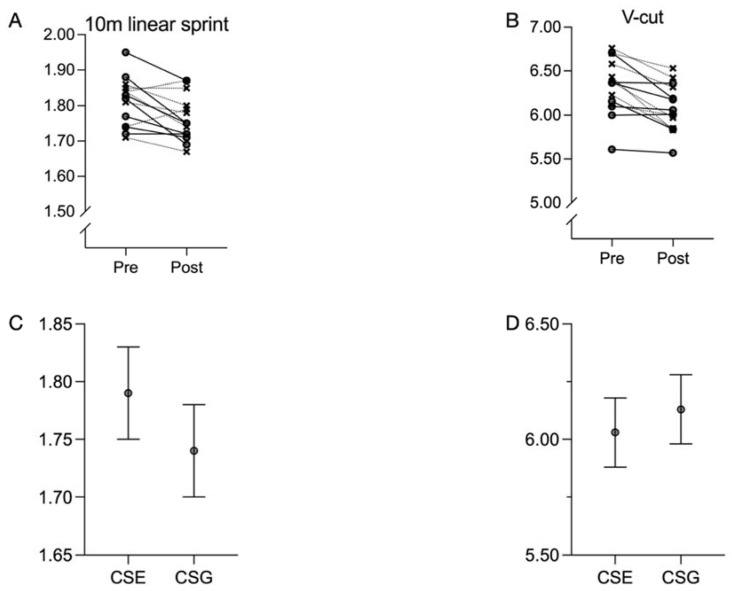
Performance scores changes for (**A**) 10-m linear sprint and (**B**) V-cut tests following both interventions. SCS group with dashed lines and cross symbols. GCS represented with solid lines a circle symbols. Comparison of adjusted mean values of both groups for (**C**) 10-m linear sprint and (**D**) V-cut tests following both interventions. SCS: specific CORE stability intervention group; GCS: general CORE stability intervention group.

The results of the Sprint and V-cut tests are shown in Figure 2. Changes observed following training in the Sprint and V-cut score tests for the entire sample and for each group are presented in Table 3.

For both tests, the same pattern was observed; there was a significant improvement for the entire sample from the pretest to the posttest, with effect sizes ranging from moderate (*d* = 0.84 (0.22, 1.45)) to large (*d* = 1.24 (0.52, 1.93)) (Table 13 and Figure 2A,B). However, no significant differences between groups were observed, with effect sizes ranging from small in V-Cut (*d* = 1.03 (−0.25, 2.30)) to moderate in Sprint (*d* = −0.56 (−1.89, 0.78)) (Table 14 and Figure 2C,D).

Following training, large significant differences were observed in the Sprint test for the GCS group (*d* = 1.46 (0.34, 2.53)) but small not-significant differences for the SCS group (*d* = 0.41 (−0.38, 1.17)). Furthermore, large significant differences following training were observed in the V-cut test for the SCS group (*d* = 1.98 (0.64, 3.28)) but moderate not-significant differences for the GCS group (*d* = 0.80 (−0.09, 1.64)).

## 4. Discussion

The objective of the present study was to analyze the effects of the level of specificity of a *core stability program* on specific actions in youth soccer players. The main findings were that the total sample significantly improved the ability to sprint and the change-of-direction maneuverability, but no significant differences were found between groups at the end of the intervention. Despite this, the magnitude of the pre- and post-training effect of the GSC group was higher than the SCS group in sprint capacity. In contrast, the magnitude of the pre- and post-training effect of the SCG group was higher than that of the GCS group in the change-of-direction maneuverability.

To the authors’ knowledge there are no studies that have examined the effect of the level of specificity of a core stability training on the performance of sport-specific actions in soccer or other sport disciplines applying the recommended criteria to increase the level of sports specificity. Despite this fact, we decided to discuss our results with studies that analyzed the effects of general CORE training interventions on sport-specific actions in soccer.

### 4.1. Linear Sprint

The results suggest significant improvements in sprint capacity in the whole sample (∆ = 2.76%, *d* = 0.84), but no significant differences were found between groups after the intervention (*d* = 1.03). In addition, the magnitude of the pre- and post-SCS training effect was lower than that of the GCS training (∆ = 1.10% vs. 4.40% and *d* = 0.41 vs. 1.46, respectively). This positive effect on sprint performance could be explained by two reasons. First, core stability plays a specific role in transferring forces to the lower extremities in sports activities such as running or hitting [2,5,8,10,13,14,15]. Second, this effect could also be explained because a *rigid and stable core* improves the speed and distal performance of the extremities [8,14,15].

These results coincide with those of Lago et al. [20]. In this study, the effects of a central musculature program on stable surfaces compared to unstable surfaces were investigated during a training period of 6 weeks (3 sessions per week) on professional indoor female soccer players. The 10-m sprint performance improved significantly in the two groups at the time of evaluation (stable condition: ∆ = 4.37%; *d* = 2.00 and unstable condition: ∆ = 5.00%; *d* = 1.13) but did not show differences between groups. The effect size obtained by our GCS was lower than the stable condition and higher than the unstable condition reported by Lago et al., while the effect size of the SCS was lower than both conditions. It should be noted that the duration of the intervention was lower, but the total number of sessions and its frequency were higher. Additionally, the exercises used had a lower level of specificity compared with our SCS group, being similar to our GCS group.

In contrast, in a similar study by Prieske et al. [21], where the changes in neuromuscular and athletic performance were investigated after training of the central musculature, with conventional core exercises and variations performed on stable surfaces compared to unstable surfaces in elite youth soccer players for a period of 9 weeks (2–3 sessions per week), no main effect was observed either at the time of assessment or between groups in the 10-m-sprint time (stable condition improvement: ∆ = 1.90%; *d* = 0.75 and unstable condition improvement: ∆ = 1.30%; *d* = 0.33). The effect size of our GCS group was higher, while the effect size of our SCS group was lower than the stable condition and similar to the unstable condition.

In a study by Doganay et al. [31] U-19 soccer players added a general CORE intervention to their usual training routine for 8 weeks (3 sessions/week, 30–35 min each session). The control group only performed their usual training routine. Both groups did not improve the 40-m linear sprint (CORE group: ∆ = 2.28%; *d* = 0.25; control group: ∆ = −1.34%; *d * = −0.14). The effect size was lower than both our groups, but it should be noted that the sprint ability was assessed with a longer sprint test. The intervention lasted the same but with a higher training frequency.

In another study [32] with U-17 soccer players, two general CORE interventions were added to the usual training routine for 12 weeks (three sessions/week with four exercises each session). One group performed stabilization core exercises (i.e., front plank, quadruped exercise, back bridge, and side bridge), while the other group performed conventional dynamic trunk exercises (i.e., sit-ups and back extension variations). The 30-m sprints were improved significantly in the stabilization core exercise group (∆ = 5.20%; *d* = 1.53) and in the conventional trunk exercises group (∆ = 5.20%; *d* = 1.43) without group*time interactions observed. The effect sizes were similar to the obtained by our GCS group and higher than our SCS group. It should be noted that the intervention lasted four weeks longer and with higher training frequency; additionally, the sprint ability was assessed with a longer sprint test.

Sever et al. [60] recruited U-19 soccer players to compare the effects of dynamic and static CORE interventions on anthropometric measurements, core stability, and field (athletic performance) tests. The interventions were added to their usual soccer training sessions for eight weeks (three sessions/week; 30 min each session). There was also a control group that only performed their usual soccer training routine. Authors reported no difference within groups or group*time interactions for the 10-m acceleration and the 30-m sprint. However, the data of the 10-m sprint test only allowed the calculation of the percentage change, which was ∆ = 1.94% for the dynamic group, ∆ = 1.25% for the static group, and ∆ = 1.21% for the control group. Despite the higher frequency of training, the percentage changes obtained were lower than our GCS group and similar to our SCS group.

In one other study [61], U-16 soccer players were recruited to examine the effects of a low-specificity CORE intervention on some motoric capabilities. The experimental group added the CORE intervention to their usual soccer training sessions for 12 weeks (two sessions/week; 30–35 min each session). The control group only performed their regular soccer training program. The authors reported significant improvements in 20-m speed tests (∆ = 0.91%; *d* = 0.19). Despite the longer duration of the intervention, the effect size was lower than ours. However, it should be noted that the participants were younger.

Forty U-19 amateur female indoor soccer players were recruited [19] to determine the effect of the core training program on speed, acceleration, the vertical jump, and the standing long jump. The intervention was added to their usual soccer training sessions for eight weeks (three sessions/week; 30 min each session). The interventions predominantly included conventional CORE exercises (i.e., frontal, lateral, and back bridges) with some standing exercises (4 out of 12 exercises). There was also a control group that only performed their usual soccer training routine. The core training group showed improvements in the 10-m linear sprint (∆ = 5.99%; *d* = 1.08), whereas the control group did not change (*p* > 0.05). Although the sex and the level of participants were different, the changes reported were higher than our SCS group and lower than our GCS group. Additionally, the intervention had more training frequency.

Finally, in a study by Hoshikawa et al. [22] the performance of the players in a linear sprint was also evaluated. In this case, the experimental group executed stabilization exercises added to the usual soccer training, while the control group only executed their usual soccer training. The authors also obtained improvements in the experimental and control groups (∆ = 1.40%; *d* = 0.56 and ∆ = 1.60%; *d* = 0.40, respectively). The effect sizes were more similar to our SCS group and lower than our GCS group, but it should be noted that the population was younger (12- and 13-year-old players), the interventions lasted longer (four sessions over 6 months), and the sprint ability was assessed with a 15-m sprint test.

### 4.2. Change-of-Direction Maneuverability

The results suggest that significant improvements were found in the change-of-direction maneuverability in the whole sample (∆ = 3.80%, *d* = 1.24), but no significant differences were found between groups after the intervention (*d* = −0.56). In addition, the magnitude of the pre- and post-SCS training effect was higher than that of the GCS training effect (5.11% vs. 2.58% and *d* = 1.98 vs. *d* = 0.80, respectively). The improvements could be conceived for the same justifications as in the sprint capacity. The greater magnitude of the pre- and post-intervention effect of the SCS group could be explained by the increase in a greater control of stabilization in dynamic and more complex actions, such as the change in direction [10].

We have not found any study that has analyzed the effects of a CS program on the ability to change direction with the V-cut test. However, Prieske et al. [21] used an agility *t*-test and found no main effect or statistically significant interaction between groups in elite youth soccer players (stable condition improved ∆ = 0.20%; *d* = 0.00 and unstable condition decreased performance by ∆ = 0.60%; *d* = 0.00).

The study of Doganay et al. [31] with U-19 soccer players obtained improvements in the CORE training group in the *t*-test (∆ = 3.73%; *d* = 0.41), while the control group did not improve any of these tests. The effect size was lower than that obtained in our study.

The study of Imai et al. [32], with U-17 soccer players, did not obtain improvements in the Step 50 test in either of the general CORE intervention groups (∆ = 1.30%; *d* = 0.55 and ∆ = 0.90%; 0.38). Both effects’ sizes were lower than those obtained in our study.

Finally, the study of Sever et al. [60], with U-19 soccer players, reported no difference within groups or group*time interactions for the 505 agility test and the Arrowhead agility test. However, the data of the tests only allowed calculation of the percentage change, which was for the 505-agility test: ∆ = 1.70% for the dynamic group, ∆ = 1.11% for the static group, and ∆ = 0.04% for the control group. For the Arrowhead agility test, it was ∆ = 0.73% for the dynamic group, ∆ = 0.37% for the static group, and ∆ = 0.39% for the control group. In all cases, our groups obtained higher percentage changes.

### 4.3. Limitations of the Study

In this study, no passive or active control group was included, which represents an added difficulty in interpreting whether the improvement in results was due to intervention programs or regular soccer training. Due to the absence of an active control group, the hypothesis that part of the observed improvements could be due to the specific soccer training should not be discarded. However, we cannot expect athletes to stop training for eight weeks in an athletic setting. Furthermore, core-strengthening exercises have been proposed to be an essential component in youth athletes’ regular conditioning program to tolerate high training loads [62,63]. The maturity status of the participants could be a confounding factor that could affect the results, and it was not assessed in the present study. Future work would be necessary in athletes with a higher training level. Although the participants in this research were similar to those in other studies [20,23,28,44,64], the sample size was small, which could lead to statistically nonsignificant results. That is why the results’ reports were focused on effect size and percentage change of the data.

### 4.4. Practical Applications

According to the effects’ sizes data, CS programs with high levels of specificity would produce higher improvements in change-of-direction maneuverability, while general CS programs, with lower levels of specificity, would produce higher improvements in sprint ability. Both general and specific CS program should be considered to improve these soccer-specific actions in youth male players. Considering the general consensus in recommending CS exercises specific to sports modalities, criteria used in this study to adjust the levels of sport-specificity of the CS programs could be interesting tools for strength and conditioning coaches.

### 4.5. Future Proposals

Future research should propose more intervention studies that analyze the real effects of the specificity of a *core stability program* on the performance of athletes. In addition, the improvements of this type of program on dynamic, complex, and specific actions at the motor level should be investigated, such as the change-of-direction maneuverability in soccer.

## 5. Conclusions

Although non-statistically significant, CS programs with high levels of specificity would be more suitable to produce improvements in change-of-direction maneuverability, while general CS programs, with lower levels of specificity, would produce higher improvements in sprint ability. Both general and specific CS programs could be used to improve sport-specific actions in youth male soccer players.

## Figures and Tables

**Table 1:** Description and methodologic criteria of SCS group’s task 1 and its progressions.

Task 1. Unilateral Skater Squat with Elastic Band	Base Task	Progression Sequence 1. Multiplanar	Progression Sequence 2. Dynamic Stabilization
**Description**	5”: explosive action and stability holding final position + 5”: repeat the same action	5”: explosive action and stability holding final position + 5”: 90° turn and stability holding final position with two points of support	5”: explosive action with jump and stability holding final position + 5”: 90° turn with jump and stability holding final position
**Methodologic criteria**			
**Standing up**	Yes	Yes	Yes
**Plane of motion**	Transverse	Transverse + sagittal	Transverse + sagittal
**Anti-rotational**	Yes. Diagonal force vector	Yes	Yes
**Unilateral**	Yes	Yes	Yes
**Movement pattern**	Jump	Jump, turn, acceleration	Jump, COD, and acceleration
**External force**	Elastic band	Elastic band	Elastic band
**Maximal speed of movement**	Yes	Yes	Yes
**Video code**	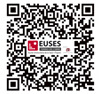	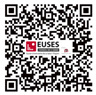	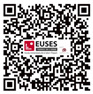

COD: change-of-direction.

**Table 2:** Description and methodologic criteria of SCS group’s task 2 and its progressions.

Task 2. Unilateral Linear Sprint with Elastic Band	Base Task	Progression Sequence 1. Multiplanar	Progression Sequence 2. Dynamic Stabilization
**Description**	5”: explosive action and stability holding final position + 5”: repeat the same action	5”: explosive action and stability holding final position + 5”: 90° turn holding final position with two points of support	5”: explosive action, acceleration, and return to starting position + 5”: 90° turn with acceleration and return to starting position
**Methodologic criteria**			
**Standing up**	Yes	Yes	Yes
**Plane of motion**	Sagittal	Sagittal + transverse	Sagittal + transverse
**Anti-rotational**	Yes. With one point of support on the floor	Yes	Yes
**Unilateral**	Yes	Yes	Yes
**Movement pattern**	Linear sprint	Linear sprint, acceleration, and turn	Linear sprint, acceleration, and COD
**External force**	Elastic band	Elastic band	Elastic band
**Maximal speed of movement**	Yes	Yes	Yes
**Video code**	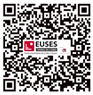	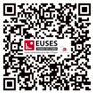	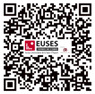

COD: change-of-direction.

**Table 3:** Description and methodologic criteria of SCS group’s task 3 and its progressions.

Task 3. Turn and 90° Pivot Shift With Elastic Band	Base Task	Progression Sequence 1. Multiplanar	Progression Sequence 2. Dynamic Stabilization
**Description**	5”: explosive action and stability holding final position + 5”: repeat the same action	5”: explosive action and stability holding final position + 5”: 90° turn and stability holding final position elevating the free leg	5”: explosive action, acceleration, and return to starting position + 5”: 90° turn with acceleration and return to starting position
**Methodologic criteria**			
**Standing up**	Yes	Yes	Yes
**Plane of motion**	Transverse	Transverse + sagittal	Transverse + sagittal
**Anti-rotational**	Yes. Diagonal force vector	Yes	Yes
**Unilateral**	Yes. Strength predominates in the front leg	Yes	Yes
**Movement pattern**	Turn and acceleration	Turn and acceleration	Turn, cod, and acceleration
**External force**	Elastic band	Elastic band	Elastic band
**Maximal speed of movement**	Yes	Yes	Yes
**Video code**	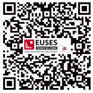	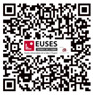	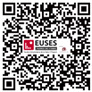

**Table 4:** Description and methodologic criteria of SCS group’s task 4 and its progressions.

Task 4. Lateral Lunge with Elastic Band	Base task	Progression Sequence 1. Multiplanar	Progression Sequence 2. Dynamic Stabilization
**Description**	5”: explosive action and stability holding final position + 5”: repeat the same action	5”: explosive action and stability holding final position + 5”: 90° turn holding final position with two points of support	5”: double lateral step and return to starting position + 5”: 90° turn with acceleration and return to starting position
**Methodologic criteria**			
**Standing up**	Yes	Yes	Yes
**Plane of motion**	Frontal	Frontal + sagittal	Frontal + sagittal
**Anti-rotational**	Yes. With one point of support on the floor	Yes	Yes
**Unilateral**	Yes	Yes	Yes
**Movement pattern**	Acceleration	Acceleration and turn	Acceleration and cod
**External force**	Elastic band	Elastic band	Elastic band
**Maximal speed of movement**	Yes	Yes	Yes
**Video code**	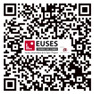	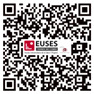	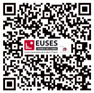

**Table 5:** Progression of the training tasks of the SCS group during the 8-week training intervention.

	Unilateral Skater Squat with Elastic Band	Unilateral Linear Sprint with Elastic Band	90° Turn with Elastic Band	Lateral Stride with Elastic Band
**Level 1. Base task**	10 reps × 10 s	10 reps × 10 s	10 reps × 10 s	10 reps × 10 s
**Level 2. Multiplanar**	10 reps × 10 s 90° turn every 5 s	10 reps × 10 s 90° turn every 5 s	10 reps × 10 s 90° turn every 5 s	10 reps × 10 s 90° turn every 5 s
**Level 3. Dynamic stabilization**	10 reps × 10 s 90° turn Dynamic jump stabilization	10 reps × 10 s 90° turn every 5 s Acceleration and return to starting position	10 reps × 10 s 90° turn every 5 s Acceleration and return to starting position	10 reps × 10 s 90° turn every 5 s Double lateral stride and return to starting position + acceleration and return to standing position

**Table 6:** Description and methodologic criteria of GCS group’s task 1 and its progressions.

Task 1. Frontal Bridge	Long-Lever Frontal Bridge	Progression 1. One-Leg Support	Progression 2. Dynamic Stabilization
**Description**	Elbows placed in front of the shoulders with the feet together	The same as the previous exercise but with one-leg support	The same as the previous exercise but with dynamic elbow flexo-extension
**Video code**	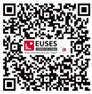	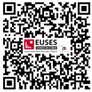	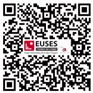

COD: change-of-direction.

**Table 7:** Description and methodologic criteria of GCS group’s task 2 and its progressions.

Task 2. Dorsal Bridge	Long-Lever Drosal Bridge	Progression 1. One-Leg Support	Progression 2. Dynamic Stabilization
**Description**	Feet in front of and higher than the knees	The same as the previous exercise but with one-leg support	The same as thw previous exercise but with dynamic hip flexo-extension
**Video code**	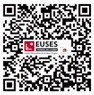	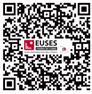	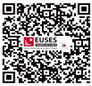

**Table 8:** Description and methodologic criteria of GCS group’s task 3 and its progressions.

Task 3. Brid-Dog	Long-Lever Bird-Dog	Progression 1. One-Leg Support	Progression 2. Dynamic Stabilization
**Description**	Hands in front of the shoulders and knees behind the hips	The same as previous exercise but with one-leg support	The same as previous exercise but with dynamic flexo-extension of the hip and the contralateral shoulders at the same time
**Video code**	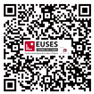	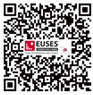	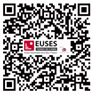

**Table 9:** Description and methodologic criteria of GCS group’s task 4 and its progressions.

Task 4. Lateral Bridge	Long-Lever Lateral Bridge	Progression 1. One-Leg Support	Progression 2. Dynamic Stabilization
**Description**	Elbow in front of the shoulder	The same as previous exercise but with one-leg support	The same as previous exercise but with dynamic flexo-extension of the upper hip and shoulder at the same time
**Video code**	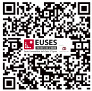	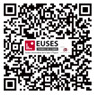	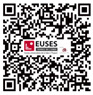

**Table 10:** Progression of the training tasks of the GCS group during the 8-week training intervention.

	Front Plank	Dorsal Plank	Bird-Dog	Lateral Plank
**Level 1. Long lever length**	10 reps × 10 s	10 reps × 10 s	10 reps × 10 s	10 reps × 10 s
**Level 2. Base of support**	10 reps × 10 s Single leg stand	10 reps × 10 s Single leg stand	10 reps × 10 s Single leg stand	10 reps × 10 s Single leg stand
**Level 3. Dynamic stabilization**	10 reps × 10 s Single leg stand Elbow flexion and extension	10 reps × 10 s Single leg stand Hip flexion and extension	10 reps × 10 s Single leg stand Knee flexion and extension + shoulder flexion and extension	10 reps × 10 s Single leg stand Knee flexion and extension + elbow flexion and extension

**Table 11:** Participants’ characteristics.

Characteristics	SCS (*n* = 7)	GCS (*n* = 7)
Age (y)	17.14 ± 0.69	16.86 ± 0.69
Mass (kg)	66.97 ± 5.05	75.09 ± 3.99
Height (cm)	1.72 ± 0.07	1.81 ± 0.05

Values are mean ± SD; y: years; SCS: specific CORE stability intervention group; GCS: general CORE stability intervention group.

**Table 12:** Reliability data.

	Pre-Test		Post-Test	
Test	CV (95% CI) (%)	ICC (95% CI)	CV (95% CI) (%)	ICC (95% CI)
Sprint_(s)_	2.89 (1.94, 3.83)	0.70 (0.31, 0.89)	1.86 (1.12, 2.60)	0.86 (0.67, 0.95)
V-Cut_(s)_	2.53 (1.57, 3.49)	0.83 (0.58, 0.94)	2.35 (1.54, 3.16)	0.87 (0.68, 0.95)

Sprint: linear sprint test of X m; V-Cut: change-of-direction maneuverability test; CV: coefficient of variation; CI: confidence intervals; ICC: intraclass correlation coefficient.

**Table 13:** Raw means and effect sizes for performance tests for the entire sample (*n* = 14) and each group SCS (*n* = 7), and GCS (*n* = 7).

Variable	Pre-Test	Post-Test	Δ (%)	MD (95% CI)	*p*	*d* (95% CI)	Qualitative Assessment
Sprint_(s)_	1.81 ± 0.07	1.76 ± 0.06	−2.76	0.05 (0.01, 0.08)	0.008 *	0.84 (0.22, 1.45)	Moderate
SCS	1.81 ± 0.06	1.79 ± 0.07	−1.10	0.02 (−0.03, 0.07)	0.318	0.41 (−0.38, 1.17)	Small
GCS	1.82 ± 0.08	1.74 ± 0.06	−4.40	0.07 (0.03, 0.12)	0.008 *	1.46 (0.34, 2.53)	Large
V-Cut_(s)_	6.32 ± 0.32	6.08 ± 0.27	−3.80	0.24 (0.13, 0.35)	<0.001 *	1.24 (0.52, 1.93)	Large
SCS	6.46 ± 0.24	6.13 ± 0.29	−5.11	0.32 (0.17, 0.48)	0.002 *	1.98 (0.64, 3.28)	Large
GCS	6.19 ± 0.35	6.03 ± 0.26	−2.58	0.16 (−0.02, 0.34)	0.079	0.80 (−0.09, 1.64)	Moderate

Data are presented in mean ± SDs. Sprint: linear sprint test of X m; V-Cut: change-of-direction maneuverability test; MD: mean difference; CI: confidence intervals; *d*: Cohen’s *d* effect size. * *p* < 0.05 pre- and post-training effect.

**Table 14:** Adjusted means and effect sizes for performance test scores between the groups.

	SCS	GCS	MD (95% CI)	*p* Tukey	*d* (95% CI)	Qualitative Assessment
Sprint(s)	1.79 (1.75, 1.83)	1.74 (1.70, 1.78)	0.05 (−0.01, 0.10)	0.082	1.03 (−0.25, 2.30)	Moderate
V-Cut(s)	6.03 (5.88, 6.18)	6.13 (5.98, 6.28)	−0.10 (−0.32, 0.13)	0.370	−0.56 (−1.89, 0.78)	Small

Score values of both groups are presented as estimated means with a 95% confidence interval. SCS: specific CORE stability intervention group; GCS: general CORE stability intervention group; *d*: Cohen’s *d* effect size.

## Data Availability

Data available on request due to restrictions, e.g., privacy or ethical restrictions.
